# Disordered gambling among people with psychotic disorders: a systematic review

**DOI:** 10.1038/s41537-023-00421-5

**Published:** 2024-01-03

**Authors:** Anoop Sankaranarayanan, Preethi Ramanathan, Rinu Mathew, Helen Wilding, David Castle

**Affiliations:** 1 Blacktown and Mt Druitt Mental Health Service, Western Sydney LHD Mental Health Service, Sydney, Australia; 2 Translational Health Research Institute, Faculty of Medicine, Western Sydney University, Sydney, Australia; 3 Senior Research Librarian, St Vincent’s Health Library Service, St Vincent’s Hospital, Melbourne, Australia; 4 Professor of Psychiatry, Centre for Mental Health Service Innovation and University of Tasmania, Hobart, Australia

**Keywords:** Psychosis, Human behaviour

## Abstract

Disorders of gambling are more common among the mentally ill, including in people with psychotic disorders. The aim of this study was to conduct a systematic review of the literature regarding the prevalence and correlates of gambling disorders in people with psychotic disorders. We systematically reviewed English-language literature through searches of six bibliographic databases, all run on 11 November 2022: Medline ALL, Embase, Emcare, APA PsycINFO, CINAHL and the Cochrane Library. Observational studies that reported the prevalence of gambling in psychotic disorders or psychosis among gamblers were included. Studies were critically appraised using the Joanna Briggs Institute Critical Appraisal Tools. Sixteen studies, including 1,116,103 participants, from across a range of settings, were included. Most studies were done on males and recruited participants with a mean age of 40 years. Most of the studies (*n* = 12) were cross-sectional, and the remaining were case control in design. Most of the studies rated fair in quality. The prevalence of gambling among psychotic population ranged from 0.32 to 19.3%, with the majority of the studies reporting rates between 6.4 and 17%. The rates were 5–25 times higher than in the general population. While there were no consistent associations found with socio-demographic indices, several studies reported an association between gambling behaviours and substance use disorder among those with psychotic illnesses. Our research suggests that clinicians should assess for comorbid gambling among those with psychotic illness, particularly in those with mood symptoms, impulsivity, and substance use disorders. Gambling can negatively impact on their financial and social situations. Future research should study specific strategies or therapies among those with comorbid gambling and psychotic disorders.

## Introduction

Gambling is “wagering something valuable on an outcome that is unpredictable^1^” and requires three elements: consideration, prize, and chance^2^. Within Australia, the Australian Productivity Commission Inquiry Report (2010) indicates that 70% of Australians participated in some form of gambling the previous year and at least 4% of the adult population gambled weekly. Australians have the highest rate of gambling losses per capita in the world. Gambling provides 11% of the state taxation revenue, the largest share of which comes from poker machines^3^. A World Health Organisation (WHO) report^4^ on gambling disorder released in 2017 states that the burden of gambling in Australia and New Zealand “is similar to that for major depressive disorder and alcohol misuse and dependence, 2.5 times more than that of diabetes, and 3 times more than drug use disorder”. High rates for gambling have also been reported from the United Kingdom (UK) where 54% of the adult population gambled in 2018 and the gambling marketed a profit of 14.2 billion pounds in 2020 [Gambling-related harms evidence review: summary - GOV.UK (www.gov.uk)] [Gambling Commission website]. The prevalence in different parts of the world is estimated to be between 0.1 and 5.8% in 2019^5^. Gambling behaviours are influenced by regulatory measures including physical restrictions (e.g., banning of specific games) and social accessibility (e.g., where certain games are socially unacceptable), as well as by cognitive accessibility (e.g., where games are difficult to comprehend) and by “substitution of games” when new games replace existing ones^6^.

Several terms have been used to describe gambling that becomes problematic. These include disordered gambling, excessive gambling, compulsive gambling, problem gambling, and pathological gambling. All are used to describe gambling-related behaviours that often lead to significant harm to self, others, and the community^7^. Extant research suggests that gambling disorders, irrespective of severity, are associated with adverse mental, physical, and psychosocial difficulties such as relationship breakdown and financial or legal problems^8^. For this reason, the term problem gambling has gained traction and usually refers to a broader spectrum of gambling behaviours that range from moderate difficulties (meeting some but not all diagnostic criteria) to extreme levels of harm that could otherwise be classified as pathological gambling. Toce-Gerstein et al.^9^ proposed placing gambling behaviours along a continuum according to the number of DSM IV symptoms manifested: risk gambling (1–2 symptoms), problem gambling (3–4 symptoms), and pathological gambling (≥5 symptoms on DSM). DSM-5 has introduced three levels of severity: mild, moderate, and severe^10^. The term pathological gambling was first introduced into the Diagnostic and Statistical Manual of Mental Disorders (DSM III) in 1980 to characterise individuals who are chronically and progressively unable to resist impulses to gamble and whose personal lives or vocational pursuits have been compromised by their gambling^11^. The different iterations of the DSM have seen a recategorization of gambling behaviours from impulse control disorders in DSM IV to addiction and related behaviours in DSM 5.

Studies from the USA, Canada and Europe typically describe a prevalence rate of 2–5% for problem gambling^1,11,12^; and 0.75–2% for pathological gambling. Similar rates were estimated from a meta-analysis of studies worldwide^13^. Problem and pathological gambling have been shown to be associated with male gender^14^, socio-economic disadvantage^15^, homelessness^16^, high rates of psychiatric comorbidity^17^, substance use^18^, suicide attempts^19^, and criminal and aggressive behaviours including felony convictions, perpetration of spouse or partner abuse, and perpetration of physical child abuse^20^.

Despite the high psychiatric comorbidity, gambling behaviours are often not assessed in clinical settings treating adults with psychiatric disorders^21^ and most individuals with gambling disorder never receive treatment^22^. Several studies have described an association between psychotic illnesses and gambling disorder^8,12,23^. This is particularly important given that people with psychotic illnesses are typically less socially inclined suggesting that other motivators or factors could drive gambling behaviour in this cohort. Furthermore, people with psychotic disorders are an already disadvantaged group in terms of low rates of employment, high rates of poverty and reliance on government disability payments^24^; gambling can have a particularly damaging impact in this context. Pullman et al.^25^ listed the gaps in literature on gambling in those with serious mental illness (SMI). Accordingly, it is important to understand the prevalence, geographical differences, and types of gambling, the instruments used, risk factors, treatment programs, and prevention strategies in this population. We believe, therefore, it is important to summarize what we know thus far, to position ourselves better in planning future research. To investigate these issues further, we systematically reviewed published studies that report an association between psychosis and disordered gambling. We aimed to summarize the rates and correlates of disordered gambling among people with psychotic illnesses. We hypothesised that the rates would be higher than seen in the general population. In keeping with reports on gambling in general, we also hypothesised that gambling disorder in psychosis would be associated with being male, younger age, lower education, and lower socio-economic status.

## Methodology

### Information sources

Publications were identified through searches of six bibliographic databases, all run on 11 November 2022: Ovid MEDLINE(R) ALL 1946 to November 09, 2022; Embase 1974 to 2022 November 09 (Ovid); Ovid Emcare 1995 to 2022 Week 44; APA PsycInfo 1806 to October Week 5 2022 (Ovid); CINAHL (EBSCOhost) and the Cochrane Library (Wiley). Reference lists of included studies were examined for additional publications. The Preferred Reporting Items for Systematic Reviews and Meta-Analyses (PRISMA) approach was followed (Page et al.)^26^. This study was registered in the INPLASY (registration number 202330108).

### Search strategy

Search strategies were developed by a medical librarian (HW) in consultation with AS and DC. Potential search terms were identified through text mining in (https://hgserver2.amc.nl/cgi-bin/miner/miner2.cgi) PubMed PubReminer using the query “pathological gambling AND (psychosis OR psychotic OR schizophrenia)”. Search terms retrieved through text mining were extensively tested for usefulness and relevance in Ovid Medline to develop the final search strategy.

**Fig. 1:**
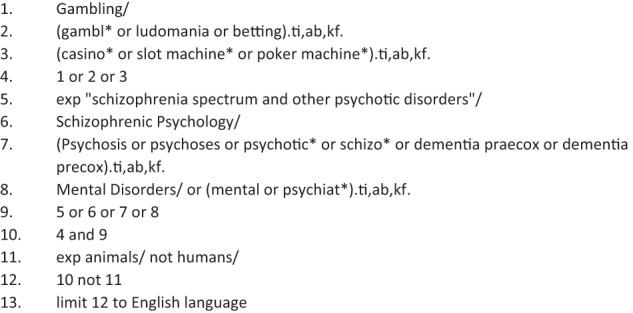
Search strategy for Ovid MEDLINE(R). Search strategy for Ovid MEDLINE(R) ALL 1946 to February 01, 2022.

Final search strategies combined the general concepts of Pathological gambling AND (Psychosis OR Schizophrenia) using a combination of subject headings and text words. In accordance with inclusion and exclusion criteria (Table 1), searches were limited to English language publications, but no date limits were applied. An initial search was developed for Ovid Medline (Fig. 1) and then adapted for other databases adjusting subject headings and syntax as appropriate (Appendix 1). Search syntax used in the Ovid databases was adapted for CINAHL (EBSCOhost) and Cochrane (Wiley) using the Polyglot Search Translator^27^.

**Table 1:** Inclusion and exclusion criteria.

Inclusion criteria	Exclusion criteria
Patient population 1. Adult patients (over 18 years and under 65 years) 2. Diagnosis of schizophrenia, schizoaffective disorder, Psychosis NOS, first episode psychosis or first episode schizophrenia, early psychosis or early schizophrenia, schizophreniform psychosis Study type 1. cross-sectional, case-control, or cohort 2. clinical trials provided they report on “pathological gambling” or “gambling behaviour”. 3. secondary analysis of databases or clinical trials Interest: reports on frequency, incidence or prevalence) of pathological gambling reports or describes correlates for pathological gambling in the population above Comparator: none or no pathological gambling Outcome: frequency or correlates. Correlates studied include socio-demographic correlates (age, gender, ethnicity, social status, occupational status, educational status, others), and clinical correlates (positive symptoms, negative symptoms, cognitive symptoms, mood symptoms, alcohol use, drug use, cigarette smoking, impulsivity, and others described in the studies).	overviews reviews perspective papers editorials case-reports case-series other diagnostic groups other age groups

### Selection process

Search results were exported to EndNote Version 20 bibliographic management software and duplicates removed by HW. In accordance with inclusion and exclusion criteria (Table 1), records were screened on publication type by HW within EndNote and excluded publication types were removed. All remaining records were loaded into Covidence systematic review software [https://www.covidence.org/] for screening on title and abstract.

Records were independently screened on title and abstract in Covidence by RM and PR. Conflicts were resolved by AS. Studies that met the inclusion criteria on title and abstract review, or that could not be excluded based on information provided in the abstract were reviewed at full text level. Full text records were assessed for eligibility by RM or PR, with conflicts resolved by AS. Studies were included if they reported rates and/or correlates of gambling disorder in people with psychotic illness and were published in English. Studies that included an admixture of diagnoses were included if data pertaining to psychotic disorders could be extracted. We were only interested in data on schizophrenia spectrum disorders (SSD) including schizophrenia, schizoaffective disorder, Psychosis NOS, first episode psychosis, first episode schizophrenia, early psychosis, early schizophrenia, or schizophreniform psychosis. We excluded information on psychotic depression or mania with psychosis but collected information on comorbid mood symptoms among those with SSD.

We limited studies in people of working age group (18–65 years). This is because there are considerable differences in gambling patterns of older adults compared to younger adults with regards to motivators for gambling, types of gambling behaviours^28^, gender differences^29^, and decision-making strategies.

Data extraction was conducted independently by any two of AS, RM, or PR. We extracted the following data: author, year, country of publication, type of study, aims, measures and tools used for exposure and outcome variables. The outcome variables of interest were prevalence of gambling in the population and in psychosis, socio-demographic correlates (age, gender, ethnicity, social status, occupational status, educational status, others), and clinical correlates (positive symptoms, negative symptoms, cognitive symptoms, mood symptoms, alcohol use, drug use, cigarette smoking, impulsivity, and others). In the case of substance use disorders (SUD), we collected all available information on the type of substance (e.g., smoking versus alcohol versus other illicit substances), extent and severity of information. The collected data were entered on a spreadsheet and compared for accuracy and completeness. The prevalence will be summarized as percentage for each study. We decided to include all potential studies.

### Study quality

Quality of included studies were assessed using the (Joanna Briggs Institute, 2017) Checklist for systematic reviews and research syntheses (Joanna Briggs Institute 2017 Critical Appraisal Checklist for Systematic Reviews and Research Syntheses (jbi.global).

Studies were rated separately by AS and PR and then consensus was reached through discussion using JBI critical appraisal tools for analytical cross-sectional studies, and case-control studies. These scales had a total of 8, and 10 items that were rated as yes, no, unclear, or not applicable. Based on the scores derived for each study type, the quality of the study was classified as ‘good’ if a study scored ≥6 out of a total of 8 for cross-sectional studies and ≥8 out of a total of 10 for case-control studies. Similarly, a study was rated as ‘fair’ if it scored 4–5 for cross-sectional studies and 5–7 for case-control studies, and ‘poor’ if it scored ≤3 for cross-sectional studies and ≤4 for case-control studies.

## Results

**Fig. 2:**
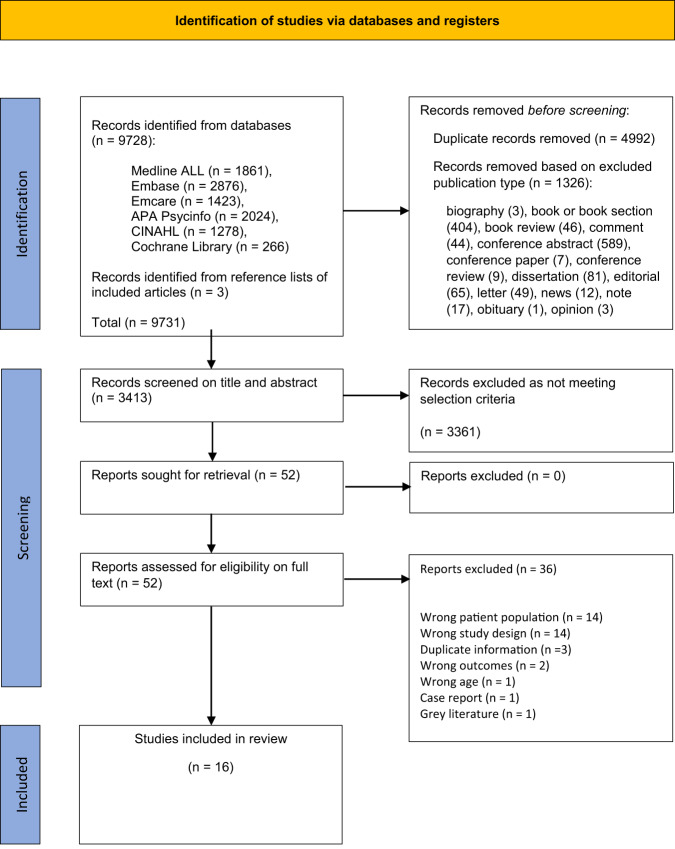
PRISMA 2020 flow diagram. From: Page et al.^26^.

In total, 9728 records were identified through database searches; 4992 duplicates were removed, and 1326 records excluded based on publication type. Full details are shown in Fig. 2.

Of the 18 articles that were identified for full text review, we excluded five studies. The excluded studies were duplicates (*n* = 4) and a qualitative study (*n* = 1) that did not provide any extractable information on gambling in people with psychotic disorders. Three additional articles were identified from hand-searches of reference lists of included studies^1,11,18^. Thus, our final review included 16 studies (please refer to Table 2 of included studies), which were based on eleven datasets. There were multiple publications from two datasets, one from the USA;^12,30,31^ and^32^ and one from Canada^33^. Most of the studies were conducted in the USA (*n* = 8), followed by Canada (*n* = 3), Australia (*n* = 2) and Spain (*n* = 2). There was one study from Italy.

**Table 2:** Summary of included articles.

Study details	Final sample	Exposure		Outcome		Frequency		Correlates	
		Diagnosis studied	Diagnostic criteria	Outcome studied	Outcome measure	In sample	In psychosis	Socio-demographic	Clinical
Aragay et al. 2012. Case-control study from Spain.	100 psychiatric inpatients ≥ 18 years were compared to 100 age and sex-matched patients with no current or past history of psychiatric illness.	Non-substance related psychiatric disorders	DSM IV TR diagnosis by two experienced psychiatrists	Gambling as No gambling (NODS 0), Risk gambling (NODS 1–2), Problem gambling (NODS 3–4), and Pathological gambling (NODS ≥ 5).	Spanish version of National Opinion Research Center DSM IV screen for gambling disorders (NODS).	9% of psychiatric group and 3% of the controls had gambling behaviour.	6/35 (17.1%) with psychosis had gambling behaviours.	Not studied	Not studied
Bergamini et al. 2018. Cross-sectional study from Italy.	900 patients attending community settings in a 6-month period, between the ages of 18–70 years and, provided they did not have organic brain syndrome.	Axis I psychiatric disorders	Mini International Neuropsychiatric Interview (MINI)	At risk gambling	Canadian Problem Gambling Index (CPGI).	At-risk gambling was seen in 48 subjects (5.3%).	The rate of at-risk gambling in schizophrenia group was 4.7%.	Younger age Male gender	Drug use, 2 or more psychiatric diagnosis, history of smoking and family history of gambling
Corbeil et al. 2021. Nested case-control study from specialist FEP clinic in Canada	219 outpatients, aged between 18–30, with diagnosis of SSD, excluding those with moderate to severe mental retardation, psychotic mood disorder, or psychosis due to general medical condition.	Schizophrenia spectrum disorders.	DSM 5 diagnosis established by the psychiatrist.	Problem gambling	Problem gambling severity index (PGSI).	Among the 219 patients with SSD, 14 patients (6.4%) had problem gambling.		Those with problem gambling were more likely to be employed and to be in a relationship	Past gambling history
Cunningham-Williams et al. 1997. Cross-sectional study from USA.	2954 St. Louisans were studied.	Mental disorders	DSM-III-based DIS, a structured diagnostic instrument for non-clinicians to assess psychiatric and substance use disorders.	Gambling.	A modified DIS criteria with definitions for pathological gambling (PG)	1250 (42.3%) had RG and 161 (5.45%) had PG	12/33 (36.36%) had RG and 6/33 had PG (18.18) Schizophrenia diagnosis had OR of 3.5 for PG versus NG	Younger age, male gender, and being divorced or separated. There was no separate data for schizophrenia.	Not studied
Desai and Potenza, 2009. Cross-sectional study from USA.	A convenient sample of 337 willing patients with SSD, receiving treatment from community mental health service	Schizophrenia spectrum disorders	Using clinician diagnosis from administrative data.	Problem and pathological gambling (PPG).	Used items from the Gambling Impact and Behaviour Study (GIBS) and the NODS, the NORC diagnostic screen for gambling.	117 (34.7%) were recreational gamblers and 65 (19.3%) had PPG. 33 (9.68%) met criteria for pathological gambling.		Those with PPG were significantly less likely to be married, report arrest, incarceration, threatening behaviours, and report spending more time with significant other in the last month.	Higher depression scores, higher alcohol scores, and more frequent outpatient contact remained significant with PPG.
Edens and Rosenheck, 2012. Nested case-control study from VA mental health users in USA.	1102846 current users of VA Mental Health Services in fiscal year 2009. Those with PG diagnosis (cases) was compared to those without (controls).	All VA mental health service users.	Clinical diagnosis from administrative data using ICD-10 codes.	Pathological gambling	ICD-10 code.	2283 of 1102846 had pathological gambling (0.21%)	265/82577 (0.32%). Schizophrenia comprised 11.6% of the gamblers (265/2283)	Middle age and female gender. Being more than 50% service connected. No separate data for schizophrenia	Having alcohol use disorder, mental illness, and presence of BPAD and PD.
Fortgang et al. 2018.	Same as for Desai and Potenza, 2009.								Substance use, especially cocaine, poly substance use, impulsivity, and delay discounting (in males).
Fortgang et al. 2020.	Same as for Desai and Potenza, 2009.								PANSS positive scores significantly associated with financial motivation to gamble. PANSS negative scores significantly associated with reduced motivation for social activity and PANSS disorganised scores with reduced motivation for company.
Granero et al. 2021 Cross-sectional study from Spain	3754 gambling disorder patients attending a specialist tertiary outpatient service for gambling disorder.	Gambling disorder	DSM-5 criteria Diagnostic questionnaire for pathological gambling	Schizophrenia	Clinician diagnosis using DSM IV and DSM V criteria	166 (4.42%) had schizophrenia.		Schizophrenia patients with gambling were significantly more likely to be males, younger, single, lower educational status, and unemployed, and of lower social status.	Comorbid schizophrenia was associated with greater severity of psychopathology (psychosis, anxiety, and depression) scores, more dysfunctional personality profile. No differences were noted in smoking, drug use, or alcohol use.
Haydock et al. 2015. Cross-sectional study from Australia.	442 participants of the Survey of High Impact Psychosis (SHIP) study from Australia. This was a community-based study of people with psychotic disorders.	Psychotic disorders.	Diagnostic Interview for Psychosis (DIP).	Problem gambling	A one-item survey followed by 9-item Problem Gambling Severity Index (PGSI) based on the CPGI.	151 (34.2%) screened positive to past-year gambling. 4.1% classified as low risk. 6.4% as moderate risk and 5.8% as problem gamblers		Male gender and leaving school with no qualifications, and long-term dependence on government financial support.	Hallucinations and medical conditions.
Kim et al. 2018a. Cross-sectional study from Brazil.	349 disordered gamblers who presented for voluntary treatment to specialist outpatient centre.	Gambling	Gambling Symptom Severity Assessment Scale (G-SAS)	Psychosis	Portuguese version of the Mini-International Neuropsychiatric Interview (MINI).	25/349 patients with gambling had psychosis (7.2%)		Females and less educated.	Impulsivity higher in disordered gamblers with psychosis Impulsivity associated with greater gambling severity. Impulsivity weakened association between psychosis and gambling.
Kim et al. 2018b. As above.	Same as above.							None reported	Current alcohol use or substance use, and greater misuse of four addictive behaviours: shopping, binge eating, prescription drugs, and caffeine.
Lesieur and Blume, 1990. Cross-sectional study from USA.	105 patients admitted to acute adult psychiatric services at a private (South Oaks) Hospital.	Psychiatric diagnosis.	Not specified	Gambling	South Oaks Gambling Screen (SOGS).	A total of 7 patients (6.66) had pathological gambling of which 4 had SSD.	4/27 of those with psychosis (15%)	None studied.	None studied.
Machart et al. 2020 Cross-sectional study from Australia.	2388 consecutive presentations to mental health clinics linked to three homeless hostels in inner city Sydney over 8.5 years.	Mental illness.	Not specified.	Problem gambling.	Defined as problem gambling if gambling was the reason for homelessness.	289 (12.1%) had problem gambling	Rate in psychotic illness was 123/1223 or 10.5% 42.6% of those who had problem gambling had a diagnosis of psychosis.	Problem gamblers were significantly more likely to be males, ever employed for more than one year, and associated being on a financial management order.	Nil
Yakovenko et al. 2018. Same as Desai and Potenza, 2009	Same as Desai and Potenza, 2009.							No significant differences between gambling groups.	Chasing, defined as tendency to continue gambling to recoup the money recently lost in gambling, is related to gambling behaviour (including severity) and substance-use behaviour.
Zimmerman et al. 2006. Cross-sectional study from USA.	1709 outpatients from a private psychiatric hospital, the Rhode Island Hospital Psychiatry.	Psychiatric diagnosis	Structured Clinical Interview for DSM IV administered by a trained rater.	Gambling	Structured Clinical Interview for DSM IV supplemented by module on pathological gambling (PG).	Lifetime prevalence for PG is 40/1709 (2.3%) and current prevalence is 12/1709 (0.7%).	55 of 1709 had psychotic disorder and the prevalence for PG was 2/55 or 3.6%.	PG associated with male gender and less education.	More lifetime axis I DSM IV disorders*, especially BPAD I, social phobia, panic disorder with agoraphobia and other impulse control disorders

Table 2 summarizes the included studies. Across the studies, there were 1,116,103 individuals, including 85,169 people with psychotic disorders and 100 healthy controls. The remaining sample had mixed diagnosis and the majority of them were drawn from people presenting to Veterans Affairs (VA) mental health services. Sample size varied from 70^23^ to 1,102, 846^1^ None of the included studies reported sample size calculations or power calculations.

The studies were conducted across a range of settings. These included first episode psychosis patients in the community^23^, inpatients^34^, community mental health patients^35^, community residents^11^, people seeking treatment to specialised centres for gambling problems^33,36^, and mental health patients attending VA and other community mental health facilities^1,12,30,31,36^.

Most studies recruited a majority of males^1,3,8,12,23,33,36,37^; only one study^18^ had a majority of females. Most studies recruited participants with a mean age of 40 years^1,11,12,33,34,36,37^ while a few had patients in their 30s^8,11^ or younger^23^. All except two studies^3,36^ reported race or country of birth^8^, with Caucasian race being predominant in all studies.

The majority of the studies (*n* = 12) were cross-sectional^3,8,11,12,18,30–33,35–37^. The remaining three studies employed a case control in design: Aragay et al.^34^ used age and sex matched healthy controls (without mental illness), while Corbeil et al.^23^ and Edens et al.^1^ used controls (who did not gamble) from the same databases as the cases. Corbeil et al.^23^ allocated four controls per case, whereas Edens et al.^1^ used all VA mental health users on their database, resulting in a case-control ratio of 1:482.

Six studies^8,12,23,30–32^ employed datasets of people with psychotic illnesses exclusively. The remaining studies included people with a range of psychiatric diagnoses. The majority of the studies used DSM-based criteria for psychiatric diagnoses^11,18,23,33–35^, while other studies relied on clinician diagnoses^3,12,30–32,37^. The diagnostic criteria were not specified in two studies^3,37^. Most studies provided separate data for prevalence of gambling disorders in people with SSD or psychosis. We speculated that such differences in rates could be related to the instruments used or to the way in which the cases and controls were recruited. For example, it has been argued that reliance on self-reports and non-use of objective measures may affect the quality of healthy controls^38^.

However, we did not find any difference in the quality ratings of studies that compared rates of gambling in cases and controls to the rates of psychosis among gamblers. In general, the studies that explored psychotic disorders among those with gambling had larger sample sizes; the larger the sample size, the smaller the errors in estimation^39^.

Most studies used validated measures to assess gambling; for example, Canadian Problem Gambling Index (CPGI)^35^, PGSI^8,23^, Diagnostic Questionnaire for Pathological Gambling^36^, National Opinion Research Centre Diagnostic Screen for Gambling Problems (NODS)^12,30–32,34^, South Oaks Gambling Screen (SOGS)^37^, The Gambling Symptom Assessment Scale (G-SAS)^33,40^. Three studies applied DSM criteria for pathological gambling/gambling disorder^11,18,36^ while another^3^ relied on patient reports.

The majority of the studies did not identify confounders but adjusted for covariates identified as significant associations on univariate analysis^1,3,8,12,23,35^ adjusted for age, substance use disorder (SUD), and cluster B personality disorder, while Fortgang et al.^30,31^ adjusted for age and gender.

The prevalence of gambling among psychotic population could be calculated from nine studies^1,3,8,12,18,23,34,35,37^. This ranged from 0.321%^1^ to 19.3%^12^, with the majority of the studies reporting rates between 6.4 to 17%^3,8,23,34,37^. The Australian studies (Haydock et al. 2015^8^ and Machart et al.^3^ reported similar rates (12.2% and 10.5% respectively).

The included studies examined several socio-demographic correlates, with inconsistent results. For example, while six studies found no association with age^8,12,18,23,33,41^, four found an association with younger age^11,35–37^, and one with middle-age^1^. More studies reported an association between male gender and gambling^3,8,11,18,34^than they did for female gender^1,33,1^. Four studies reported an association between lower education and gambling^8,18,33,36^, while four did not^11,12,23,35^ and four did not study the association^1,3,34,37^. None of the studies that were predominantly or exclusively of people with psychotic illnesses^8,12,23^, found any consistent associations with age, gender, employment, educational achievement, or relationship status. Two studies found an association between gambling and criminal offences^12,23^.

Regarding correlates with clinical variables, several studies found evidence of significant association between gambling behaviours and SUD^8,12,18,30,35^. Other studies demonstrated associations with more severe psychotic symptoms^36^, positive symptoms^8,31^, negative symptoms^31^, depression^12,31,36^, impulsivity, binge eating^33^, smoking and a family history of gambling^35^, psychiatric comorbidity^1,18,35^, and past gambling history^23^. However, most of the studies did not explore for associations between gambling and positive, negative, or cognitive symptoms, or depressive symptoms.

In view of the heterogeneity, we undertook sub-group analyses to study whether the results would be different if we studied results based on the diagnoses studied (schizophrenia, psychosis, or schizophrenia spectrum disorder versus those with mental illness). We also studied whether the criteria to study or screen for gambling had an impact on the results.

In our first analysis, we found that six studies^8,12,23,30–32^ were done among people with psychotic disorders. Of these, four studies^12,30–32^ were reports based on the same dataset. Two studies^8,23^ rated good on qualitative analyses as they used valid measures to diagnose psychosis, and both used PGSI to measure gambling and adjusted for confounders (cluster B personality disorders, SUD, and age). They found similar rates for problem gambling (5.8% in ref. ^8^ and 6.4% in ref. ^23^) but very different correlates; while male gender, lower education, and dependence on financial supports and hallucinations were found to be associated with gambling in Haydock et al.^8^. Corbeil et al.^23^, reported association with being employed, being in a relationship, and past gambling history.

We then examined pathological gambling in those with psychotic disorders, studied as part of a broader sample that also included people with other mental illnesses. We did this by separating them into studies done among inpatients^34,37^ and those in the community/outpatients^3,11,18,35^. These studies demonstrated greater heterogeneity in the screening instruments or criteria to determine gambling behaviours (for e.g., defining gambling as problem gambling if associated with homelessness^3^ to CPGI^35^, to SCID for DSM IV^18^). In general, the rates were lower in studies done among community patients (3.6% among people with psychosis^18^, and 4.7% in schizophrenia patients^35^. Machart et al.^3^ reported a rate of 10.5% for problem gambling among those with psychosis; however, they used a different criterion for problem gambling). When we removed this study^3^ from the analysis (as it did not specify how psychosis was defined), it led to more consistent rates of 3.6–4.7%.

Cunningham-Williams et al.^11^ used DSM III based criteria and reported a rate of 18.18% for pathological gambling among those with psychosis; they were the only study that also studied confounders (age, gender, ethnicity, SUD, and antisocial personality disorder). The rates were higher for inpatients; 17.1% had problems with pathological gambling in those with SSD based on DSM IV NODS screening^34^, whereas 15% was reported in those with psychosis based on the DSMIII SOGS screening^37^. Both studies were fair in quality and neither identified or adjusted for confounders or correlates. Although Lesieur and Blume^37^ also did not specify how psychosis was diagnosed, removing this study did not alter the rates of gambling among psychotic inpatients.

Table 3 summarizes the quality ratings. Eight studies were rated as ‘fair’ (*n* = 8)^1,18,30–32,34,36,37^, six as ‘good’^8,11,23,33,35,40^, and two as ‘poor’^3,12^. The main weaknesses included lack of information on or not adjusting for confounders, not using valid instruments to measure exposure or outcome variables, and poor definition of selection criteria of sample studied.

**Table 3:** Qualitative analysis.

Study	JBI tool used	Score received	Strengths	Limitations
Aragay et al. 2012	Checklist for case-control study	5/10 Fair	Groups were comparable and matched appropriately; the same criteria were used for identification of cases and controls. Used standard, valid, and reliable way to measure outcomes in cases and controls. Used appropriate statistical analysis.	The authors relied on clinician diagnosis and did not employ the same methods in cases and controls. No information on duration of illness. Did not identify confounders or report statistical methods to deal with confounders.
Bergamini et al. 2018	Checklist for cross-sectional studies	6/8 Good	Descried the subjects, and setting, the inclusion criteria. Used valid and reliable measures for psychiatric diagnoses. Outcome measured in a valid and reliable way. Appropriate statistical analyses used.	Did not identify or describe strategies for confounders.
Corbeil et al. 2018	Checklist for case-control study	9/10 Good	This was a nested case-control study, and the groups were comparable and matched appropriately; the same criteria were used for identification of cases and controls. Same method to diagnose SSD in cases and controls. Described confounders and strategies to deal with confounding. Gambling was assessed in a standard, valid, and reliable way. Exposure period of interest was long enough, and appropriate statistical analysis was used.	Diagnosis of schizophrenia/SSD was not done in a standard, valid or reliable way
Cunningham-Williams et al. 1997	Checklist for cross-sectional studies	6/8; Good	Authors described the sample reasonably well, used valid tools for diagnosis, but amended the criteria for gambling. They identified and adjusted for confounders,	Inclusion criteria not clear Tool for gambling not clear.
Desai and Potenza, 2009	Checklist for cross-sectional studies	2/8 Poor	The authors used valid measures for gambling	They did not define or describe their sample, the selection criteria, how the diagnosis of SSD was made/reached or measured. They did not identify confounders.
Edens and Rosenheck 2012	Checklist for case-control study	5/10 Fair	The authors had a large nationally representative sample of VA personnel and employed multivariate analysis.	Their large sample size could have magnified small differences. They relied on clinician diagnosis for mental illness or gambling.
Fortgang et al. 2018	Checklist for cross-sectional studies	5/8 Fair	The authors had a reasonable sample size to undertake analysis to examine the association between substance use, impulsivity, and delay discounting in gambling.	They did not use valid tools or diagnostic criteria for schizophrenia.
Fortgang et al. 2020	Checklist for cross-sectional studies	5/8 Fair	The authors had a reasonable sample size to analyse the impact of 5-factor model of psychosis on motivators for gambling.	They did not use valid tools or diagnostic criteria for schizophrenia. Too many analyses.
Granero et al. 2021	Checklist for cross-sectional studies	5/8 Fair	Sample well defined Setting well defined Exposure measure using reliable and valid tool.	Over long period (2005–2020) meaning different criteria. Used clinician diagnosis for schizophrenia and gambling. No confounders identified
Haydock et al. 2015	Checklist for cross-sectional studies	6/8 Good	Good sample size, used valid instruments, described the sample reasonably, and adjusted for confounders	Did not identify confounders apriori and used screening instruments
Kim et al. 2018	Checklist for cross-sectional studies	6/8 Good	Describes sample, used valid and objective measures, used appropriate statistical measures	Does not describe the inclusion or selection criteria, not identified confounders or dealt with strategies to adjust for confounding. Sample selected over a long time period (9 years)
Kim et al. 2018b	Checklist for cross-sectional studies	6/8; Good	Described sample and selection criteria, used valid and objective measures and appropriate statistical methods	Did not identify confounders or report strategies to adjust for confounding.
Lesieur and Blume, 1990	Checklist for cross-sectional studies	4/8; Fair	Described the sample and selection criteria and used a valid measure for gambling	Small sample size with small cell size for individual conditions and gambling; relied on clinician diagnosis for psychosis. No confounders studied or adjusted for in statistics.
Machart et al. 2020	Checklist for cross-sectional studies	3/8; Poor	Convenience sample of homeless mentally ill; described the sample and defined problem gambling	Study done in homeless population (limiting generalisability), without using valid and reliable tools but relying on patient reports for gambling and history, broad definition for problem gambling, no confounders identified but undertook logistic regression analysis of identified associations, and study sample recruited over a long period (2008–2016). Convenience sampling. What all does psychotic illness include? The authors do not discuss their results; while multivariate analysis shows that the association with mood disorders loses significance and shows a trend for psychosis, they discuss that mood disorders are associated with problem gambling.
Yakovenko et al. 2018	Checklist for cross-sectional studies	5/8-Fair	Inclusion criteria Setting and subjects Outcome measured Stats used	Different time periods No confounders
Zimmerman et al. 2006	Checklist for cross-sectional studies	4/8-Fair	Inclusion criteria Valid tool for mental illness Outcomes measured in valid way Appropriate statistics	Did not identify or manage confounders.

## Discussion

As far as we are aware, this is the first attempt to systematically review the literature on the prevalence and correlates of disordered gambling amongst people with psychotic disorders. Further, research suggests that people with gambling behaviours that do not fulfil stringent criteria for pathological gambling also have significant disability and distress^8^.

We identified a total of sixteen studies that were mostly fair to good in quality and published from the Western world. These studies reported a broad prevalence of 0.32–19.3% for gambling among people with psychotic illnesses, which is very similar (but slightly higher) to rates reported in those with bipolar disorders (10.6–13.3%)^42,43^ and depressive disorders (9.4–12.5%)^44,45^. The rates were around 4–6% in studies conducted specifically in people with schizophrenia or SSD but varied more among mixed samples; similarly, in general, the rates were higher for inpatients (around 15–16%) compared to community-based samples. Despite the heterogeneity in the study methodologies, diagnoses, and outcomes, most studies concur that the co-occurrence of gambling and psychosis is common, and the rates are between 5 and 25 times higher than in the general population. Only one study^35^ reported rates similar to that in the general population. Interestingly, however, this research group published a second study that compared problematic gambling rates in attendees of mental health services (both inpatient and community mental health) with a broad range of psychiatric diagnoses (excluding primary SUD) to those in the general population^46^ and concluded that rates were significantly higher in psychiatric patients. However, the two samples (psychiatric patients and the control population) were recruited at two different time points.

Thus, our first hypothesis—that gambling behaviours are more prevalent among people with psychotic illnesses than in the general population—was largely confirmed.”

The variance in rates of problematic gambling reported across the studies reviewed here, could reflect, inter alia, differences in how the severity of the gambling behaviours were identified and defined. For example, Edens et al.^1^ reported low rates based on clinical diagnosis while Desai et al.^12^ used a screening instrument to identify gambling behaviours and found substantially higher rates. Amongst studies assessing rates of schizophrenia amongst people with identified gambling disorders, rates were between 3.9%^11^ and 7.2%^33^. In general, those with gambling disorders had greater severity, impulsivity, binge eating, higher rates of substance use, and criminal offences^12^. At the same time, the severity of PG may be influenced by a host of factors, including but not limited to the method of diagnosis.

Studies in the general population have consistently shown association between certain socio-demographic predictors and pathological gambling. For example, gambling is more prevalent among single, younger males from lower socio-economic strata. Such associations were inconsistently reported in the studies reviewed here. Also, even in those studies that did find such associations, these were not specifically reported for those with psychotic disorders. Aragay et al.^34^ reported that a significantly larger number of gamblers with psychotic disorders were men, had no stable partner, and had a substance use disorder, particularly cannabis use. In contrast, Edens et al.^1^ reported an association between gambling and female gender, middle age, and higher socio-economic status, and Kim et al.^33^ also reported higher prevalence in females. Corbeil et al.^23^ found that being in a relationship was associated with gambling behaviours, whereas Vita et al.^46^ reported that being married was protective against high-risk gambling. Substance use was the commonest association reported in most studies^8,12,32,34,35^. This means that our second hypothesis was only partially confirmed.

Several reasons and hypotheses have been advanced to explain the higher prevalence of gambling behaviours in people with psychotic illnesses. One theory links gambling behaviour with perturbations of dopaminergic and serotonergic systems that impact the brain’s reward systems^8,33,34^. Of interest is the fact that the dopamine D2/D3 partial agonist aripiprazole is associated with an increased odds (OR: 15.2; 95% CI 2.1–670.5) for gambling behaviours^23^. Similar mechanisms have been postulated in gambling behaviours in Parkinson’s disease patients, but available evidence does not fully explain the role of dopaminergic mechanisms in gambling disorder^36^.

The association between psychotic symptoms and gambling is less clear. While Haydock et al.^8^ reported an association between problem gambling and positive symptoms of schizophrenia, Desai et al.^12^ did not find such an association. On the other hand, recognition of an association between gambling and depressive symptoms^3,37^ have led to the suggestion that gambling may be a compensatory strategy to reduce negative affect or dysphoric mood. An alternative explanation is that gambling could lead to financial loss and therefore dysphoric mood. People with psychotic disorders are at high risk of SUD, which is also commonly associated with gambling behaviours^3,8,37^. This could mean that there is a multiplicative effect or an effect on inhibition and reward pathways (primary ventral striatum and medial prefrontal cortex) that predisposes to gambling^36^. This association could also be related to the fact that opportunities to gamble are frequently co-located in venues serving alcohol.

Yakovenko et al.^41^ undertook a qualitative study of eight individuals with comorbid schizophrenia and gambling to understand the motivations for gambling. Perhaps counter-intuitively, they found that social engagement and being around people were reasons for gambling, suggesting that this could be a strategy to avoid the social void that many people with schizophrenia experience. Haydock et al.^8^ suggested that gambling behaviours may be an offshoot of occupational deprivation and high levels of unstructured time, limited engagement in meaningful occupations, and accompanying social isolation and boredom. On the other hand, Fortgang et al.^30^ reported reduced sensation seeking in schizophrenia and opined that this motivation may be less relevant to gambling behaviours in individuals with schizophrenia, than people without schizophrenia.

Multiple studies have emphasised an association between increased impulsivity and gambling^8,12,30,33^. Impulsivity is also associated with SUD, negative affect, and greater severity of gambling. There is also a tendency for higher comorbid shopping, binge eating, prescription drug use, caffeine use^33^, nicotine and substance dependence, and being overweight^3^. Studies have also investigated psychological phenomena associated with impulsivity and gambling such as delay discounting (which basically means that the subjective value of a future reward is less than that of an immediate reward)^47^ and chasing behaviours (term to describe the phenomenon when people continuing to gamble with increased wagers after a sequence of losing bets, to make good the loss)^48^. These studies found higher delay discounting levels in men^30^, and chasing behaviours^32^ were associated with greater impulsivity and gambling behaviours in psychotic population. All of this suggests greater comorbidity between gambling and other forms of “addiction” and higher impulsivity. Difficulties in emotion regulation and cognitive biases around gambling activities have also been put forth as explanations for comorbid gambling and psychosis^36^.

In the studies reviewed here, gamblers with psychosis were generally more likely to report an earlier age of onset^35^, more frequent gambling and spending more hours gambling per week^33^, as well as greater severity of symptoms and higher utilisation of outpatient mental health services^12^. Individuals with psychosis and disordered gambling may represent a uniquely vulnerable clinical population as they have important commonalities in underlying brain pathology and clinical symptomatology that occur across both disorders^49^, which may manifest in increased risk of poly-comorbidity and symptom severity among this dual disorder population. However, there are contradictory findings. For example, the typical association seen between gambling behaviours and younger age, lower education, or lower socio-economic status among the general population is not consistently seen in people with psychosis. In fact, there is at least one study that reports increased impulsivity among females^33^, suggesting important gender differences among those with psychosis that is not seen in the general population. Other factors associated with gambling in the general population such as higher rates of suicidality have also not been consistently reported among those with SSD and this could be because of higher base rates among those with SSD in general. Nevertheless, this group with dual gambling and psychotic disorders requires intervention. Evidence suggests that strategies such as cognitive behavioural therapy (CBT)^50^ and working memory training^33^ are beneficial. There may be a role in targeting impulsivity^33^.

The results presented here need to be seen in the light of important limitations. Although our search strategy was systematic and broad, we only included articles published in English, and all the studies were from Western countries, mostly the USA and Canada. This is important because, among those from non-Western background, culture influences gambling behaviours, the type of gambling that are approved or punished, and/or help seeking^51^. The absence of date limits in study selection introduces challenges, considering the changes in diagnostic criteria for both psychotic and gambling disorders over the years. There was substantial methodological heterogeneity, including in the diagnostic groups studied (mixed diagnoses versus psychotic disorders versus schizophrenia spectrum disorders or first episode psychosis); methods of recruitment (inpatient, versus outpatient, versus general population); and how gambling was assessed and defined (single question or patient reports versus screening instruments versus diagnostic criteria). Differences in key aspects such as symptom duration, the presence of mood symptoms, and illness stage can introduce heterogeneity into the sample, complicating the interpretation of the results. Factors such as severity of disorders, sample in treatment and treatment effectiveness, impact of treatment (e.g., dopamine agonists), social and environmental factors, and individual personality traits can all influence gambling behaviour and are important confounding factors.

The studies employed varied definitions for gambling behaviours and some authors collapsed multiple categories (such as moderate and high-risk categories). Further, the majority of the studies did not identify or adjust for confounders and employed modest sample sizes. The included studies also varied considerably in sample sizes; the number of patients with psychosis or schizophrenia spectrum disorders ranged from 25^33^ to 82577^1^.

There is a need for further research, preferably longitudinal studies, to clearly delineate how gambling begins and progresses among people with a psychotic illness, and to determine factors that increase vulnerability to problematic gambling amongst this group of individuals (e.g., age, gender, medication (aripiprazole), substance use, impulsivity, personality disorder or attributes). There is also a need for biological research to assess brain changes or neuropsychological or neurocognitive attributes that are associated with gambling behaviours in people with schizophrenia. Finally, given the financial and social travails all too often associated with gambling and psychosis, further research should investigate treatment modalities that can assist people with schizophrenia desist from, or at least moderate, gambling behaviours.

## Conclusions

There is a high prevalence of gambling among people with psychotic disorders. Comorbid gambling can worsen the financial and social disadvantages in this particularly vulnerable population. Specific treatment strategies such as CBT are effective in this population while other strategies such as working memory training or medications have not been studied in those with psychotic illness. There are important gaps in knowledge, which calls for further research to improve identification, management, and prognosis for gambling disorders among this group of patients.

### Supplementary information

Appendix
