# Supplementary material for: Disordered gambling among people with psychotic disorders: a systematic review
Source: Schizophrenia (Heidelb). 2024 Jan 3;10(1):4. doi: 10.1038/s41537-023-00421-5 (PMC10851698; doi:10.1038/s41537-023-00421-5)
Supplement: Supplementary file 1 — Appendix [file 41537_2023_421_MOESM1_ESM.docx]

**Search strategies**

**Ovid MEDLINE(R) ALL 1946 to February 01, 2022**

1. Gambling/
2. (gambl* or ludomania or betting).ti,ab,kf.
3. (casino* or slot machine* or poker machine*).ti,ab,kf.
4. 1 or 2 or 3
5. exp "schizophrenia spectrum and other psychotic disorders"/
6. Schizophrenic Psychology/
7. (psychosis or psychoses or psychotic* or schizo* or dementia praecox or dementia precox).ti,ab,kf.
8. Mental Disorders/ or (mental or psychiat*).ti,ab,kf.
9. 5 or 6 or 7 or 8
10. 4 and 9
11. exp animals/ not humans/
12. 10 not 11
13. limit 12 to english language

**Embase 1974 to 2022 February 01 (Ovid)**

1. gambling/ or pathological gambling/
2. (gambl* or ludomania or betting).ti,ab,kf.
3. (casino* or slot machine* or poker machine*).ti,ab,kf.
4. 1 or 2 or 3
5. psychosis/ or acute psychosis/ or affective psychosis/ or brief psychotic disorder/ or childhood psychosis/ or depressive psychosis/ or endogenous psychosis/ or manic psychosis/ or exp paranoid psychosis/ or puerperal psychosis/ or exp schizophrenia/ or schizophrenia spectrum disorder/ or schizoaffective psychosis/
6. (psychosis or psychoses or psychotic* or schizo* or dementia praecox or dementia precox).ti,ab,kf.
7. *mental disease/ or (mental or psychiat*).ti,ab,kf.
8. 5 or 6 or 7
9. 4 and 8
10. exp animal/ not human/
11. 9 not 10
12. limit 11 to english language

**Ovid Emcare 1995 to 2022 Week 4**

1. gambling/ or pathological gambling/
2. (gambl* or ludomania or betting).ti,ab,kf.
3. (casino* or slot machine* or poker machine*).ti,ab,kf.
4. 1 or 2 or 3
5. psychosis/ or acute psychosis/ or affective psychosis/ or brief psychotic disorder/ or childhood psychosis/ or depressive psychosis/ or endogenous psychosis/ or manic psychosis/ or exp paranoid psychosis/ or puerperal psychosis/ or exp schizophrenia/ or schizophrenia spectrum disorder/ or schizoaffective psychosis/
6. (psychosis or psychoses or psychotic* or schizo* or dementia praecox or dementia precox).ti,ab,kf.
7. *mental disease/ or (mental or psychiat*).ti,ab,kf.
8. 5 or 6 or 7
9. 4 and 8
10. exp animal/ not human/
11. 9 not 10
12. limit 11 to english language

**APA PsycInfo 1806 to January Week 5 2022 (Ovid)**

1. gambling/ or gambling disorder/

2. (gambl* or ludomania or betting).ti,ab.

3. (casino* or slot machine* or poker machine*).ti,ab.

4. 1 or 2 or 3

5. exp psychosis/

6. (psychosis or psychoses or psychotic* or schizo* or dementia praecox or dementia precox).ti,ab.

7. mental disorders/ or (mental or psychiat*).ti,ab.

8. 5 or 6 or 7

9. 4 and 8

10. (animal not human).po.

11. 9 not 10

12. limit 11 to english language

**CINAHL (EBSCOhost)**

1. (MH "Gambling")
2. gambl* OR ludomania OR betting
3. casino* OR slot machine* OR poker machine*
4. S1 OR S2 OR S3
5. (MH "Psychotic Disorders+")
6. psychosis OR psychoses OR psychotic* OR schizo* OR dementia praecox OR dementia precox
7. (MH "Mental Disorders") OR mental OR psychiat*
8. S5 OR S6 OR S7
9. S4 AND S8
10. (MH "Animals+") NOT (MH "Human")
11. S9 NOT S10 [Limiters – English language]

**Cochrane Library (Wiley)**

1. [mh ^Gambling]
2. (gambl* OR ludomania OR betting):ti,ab
3. (casino* OR slot machine* OR poker machine*):ti,ab
4. #1 OR #2 OR #3
5. [mh "schizophrenia spectrum and other psychotic disorders"]
6. [mh ^"Schizophrenic Psychology"]
7. (psychosis OR psychoses OR psychotic* OR schizo* OR "dementia praecox" OR "dementia precox"):ti,ab,kw
8. [mh ^"Mental Disorders"] OR (mental OR psychiat*):ti,ab,kw
9. #5 OR #6 OR #7 OR #8
10. #4 AND #9

**Clinicaltrials.gov**

Psychosis and gambling

<https://clinicaltrials.gov/ct2/results?cond=Psychosis&term=gambling&cntry=&state=&city=&dist=>
